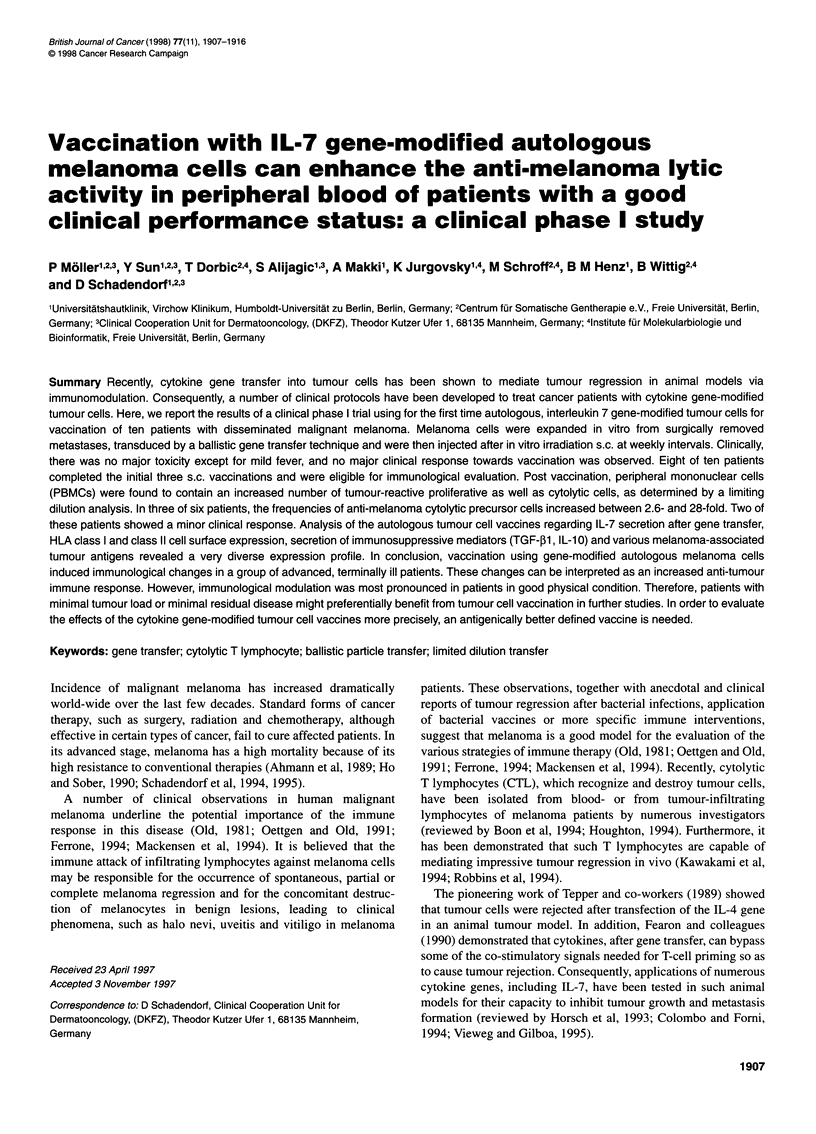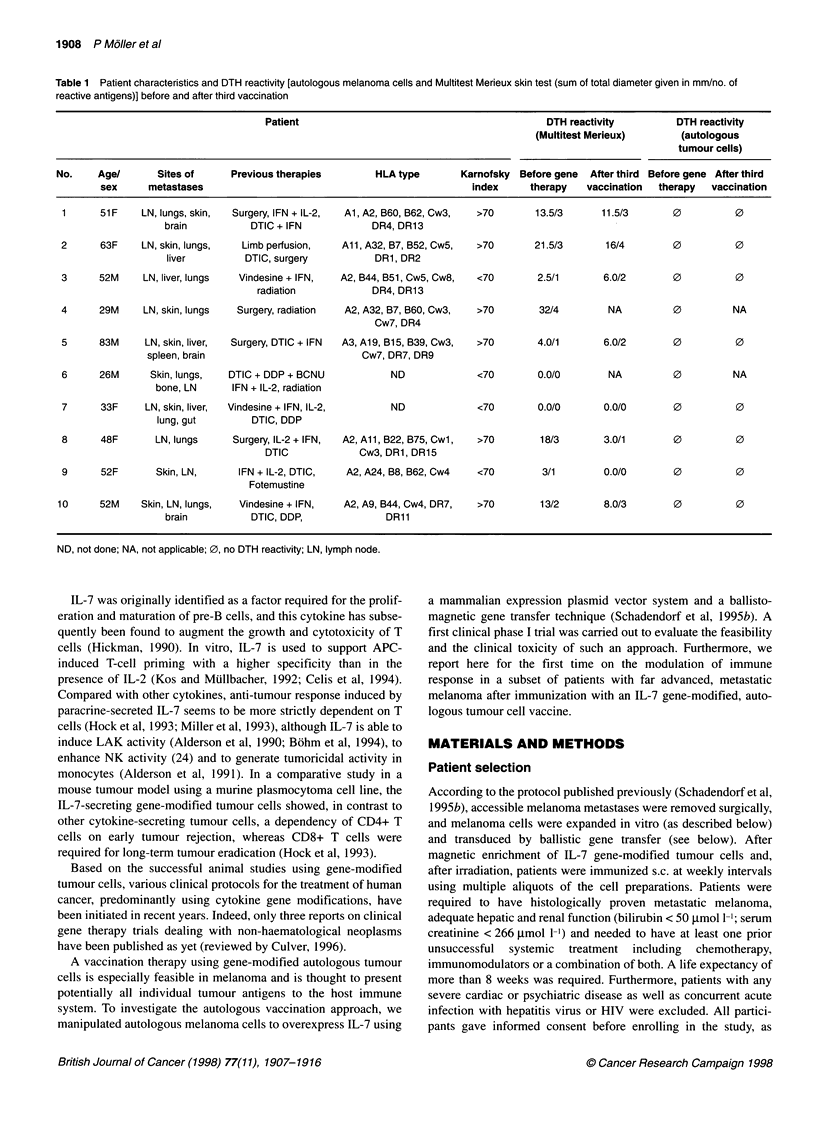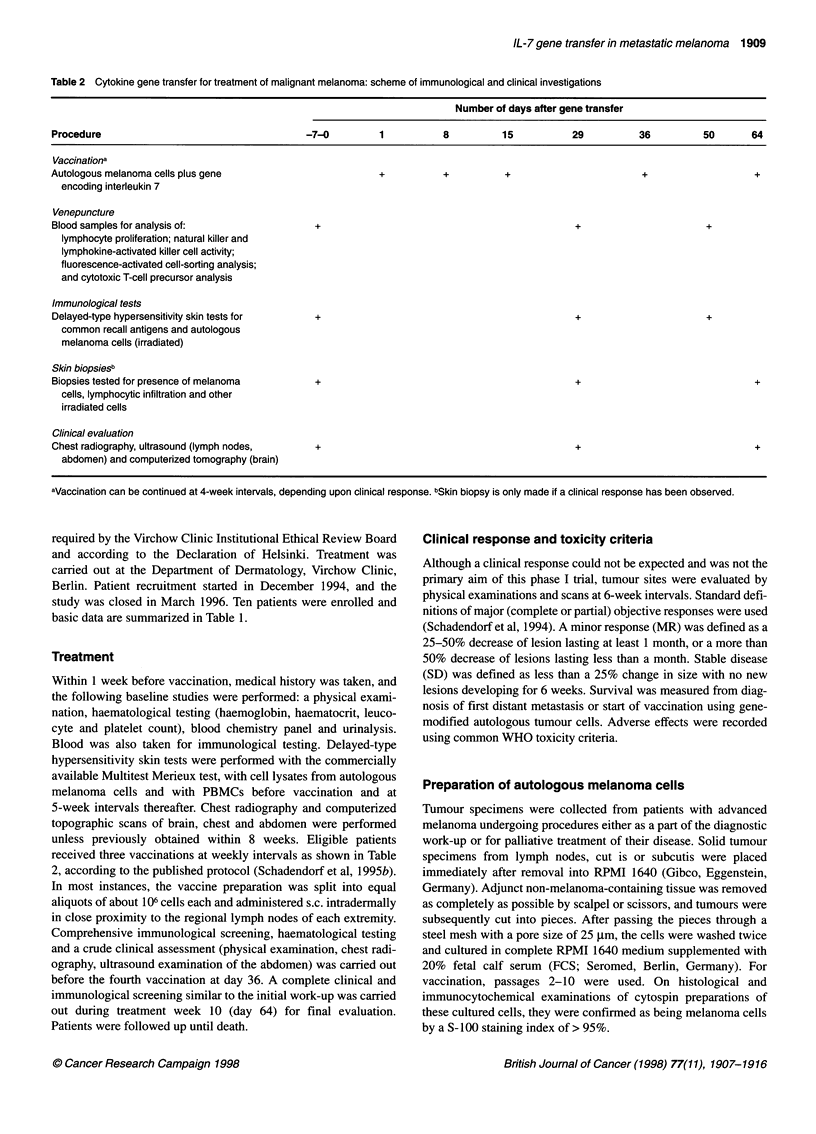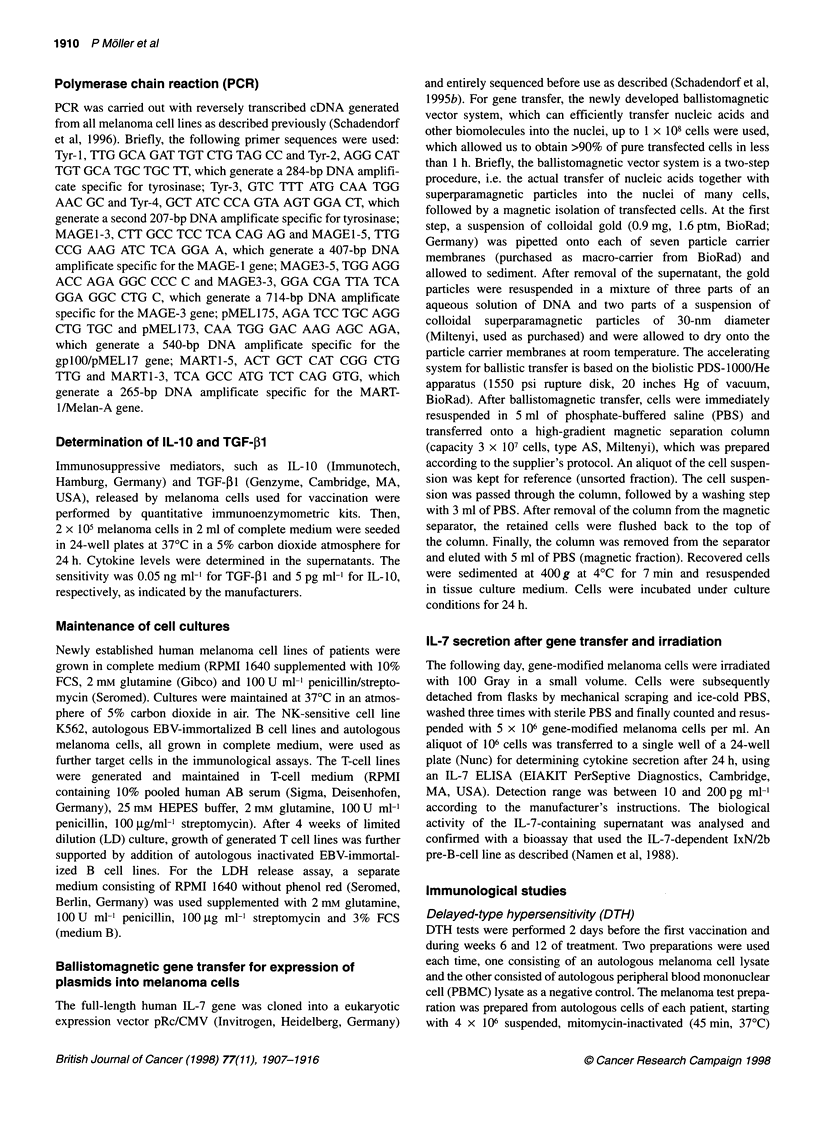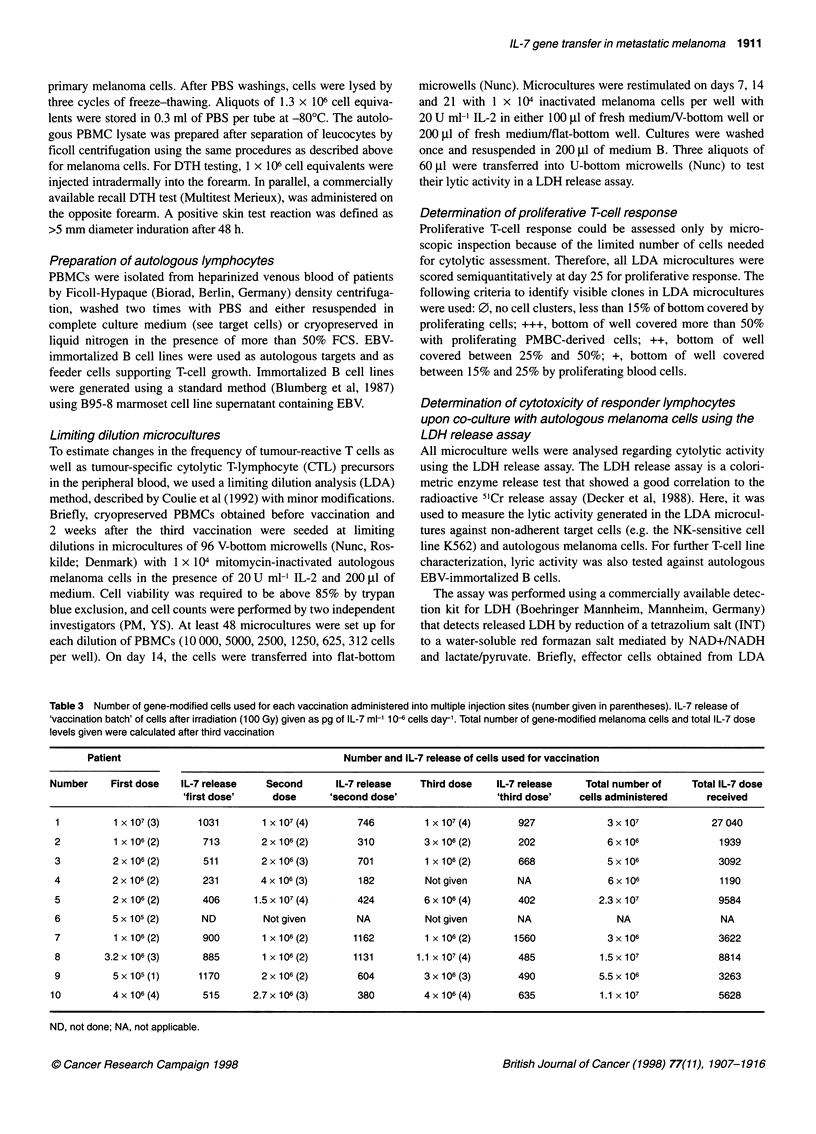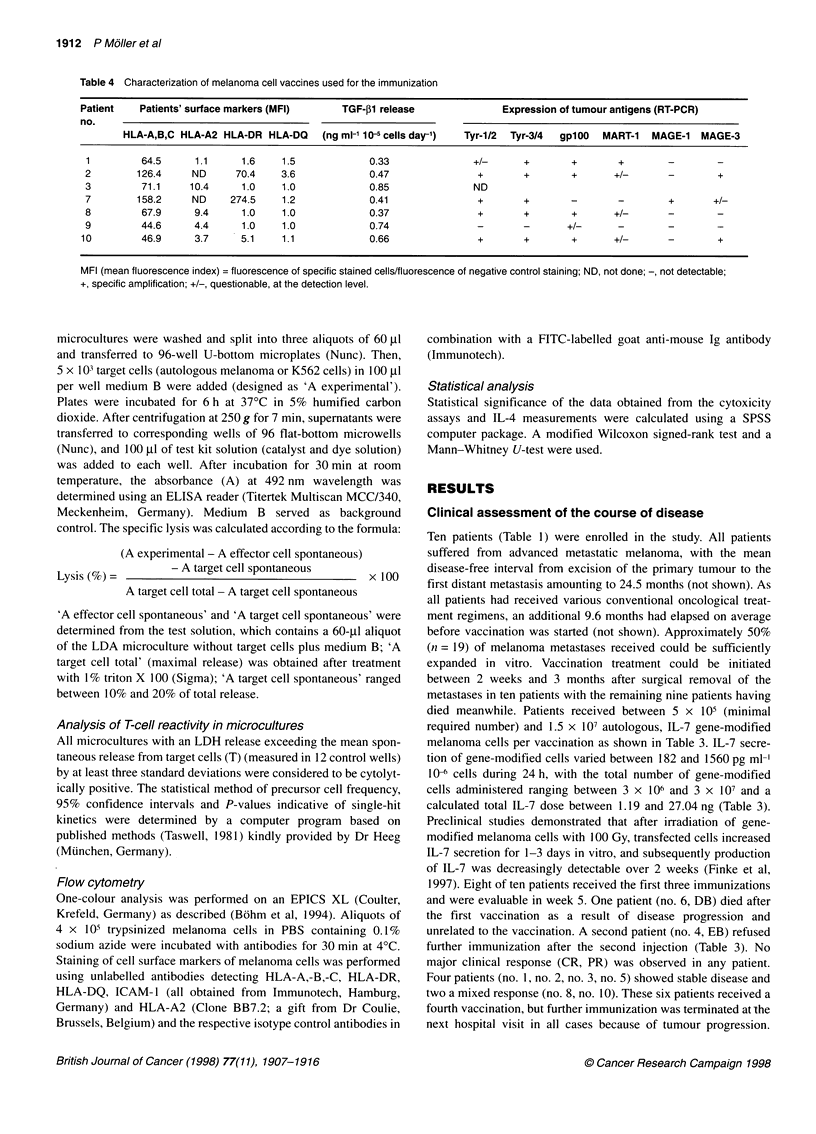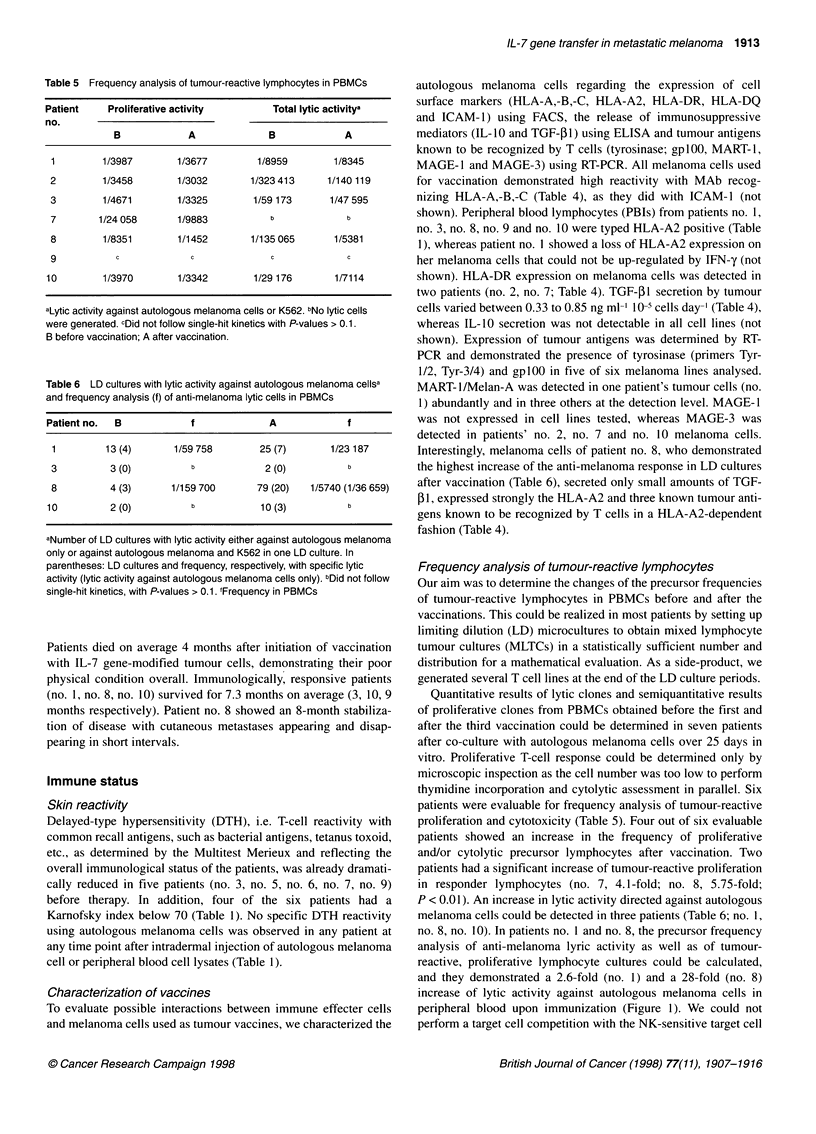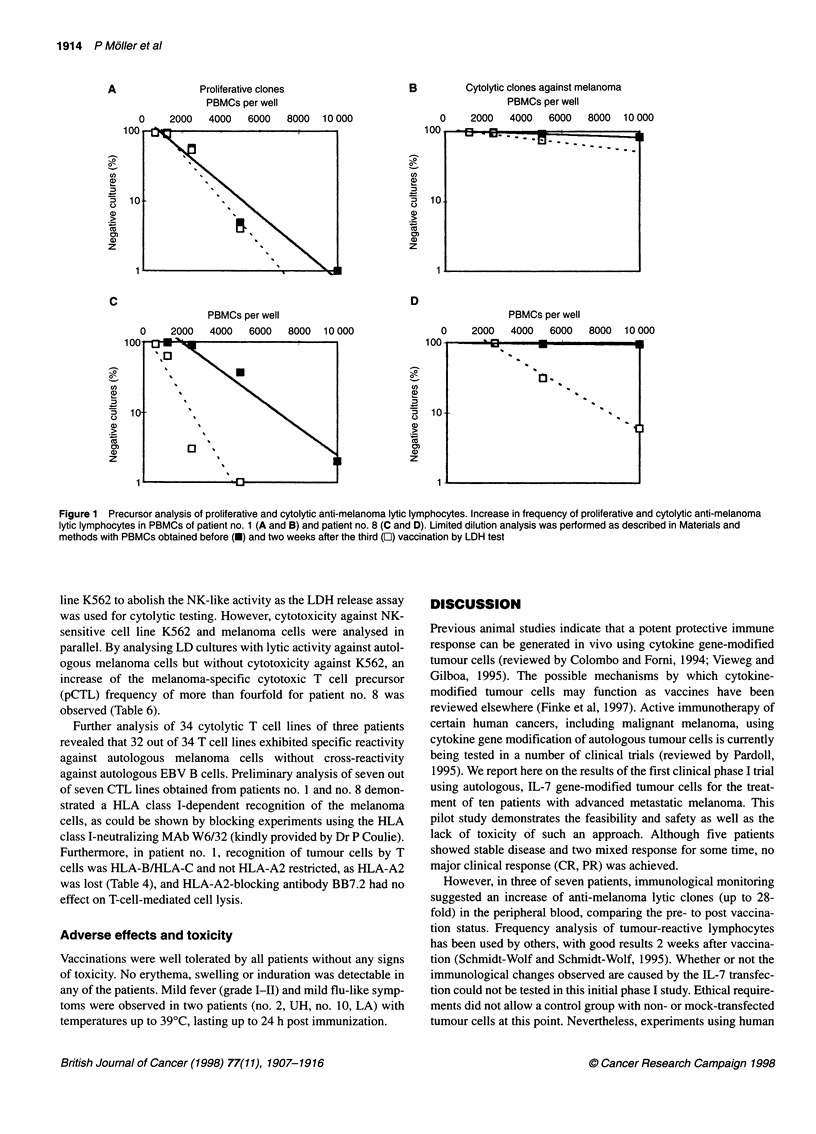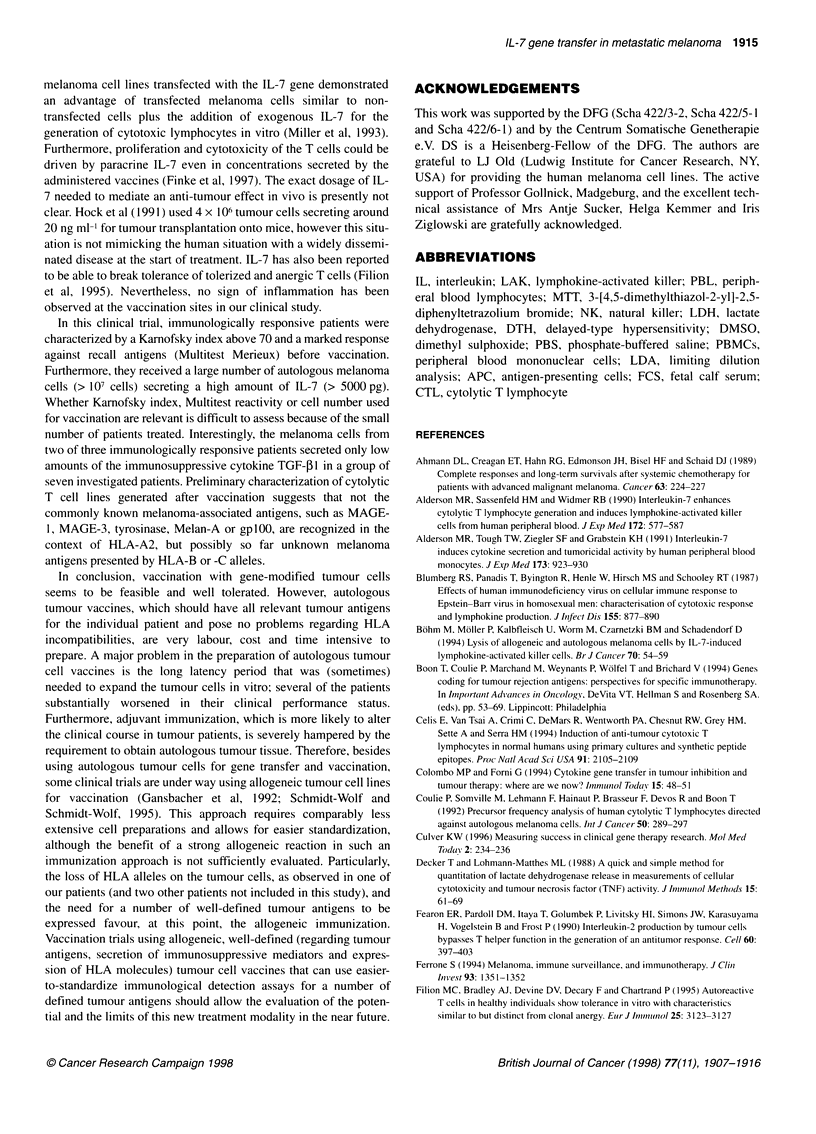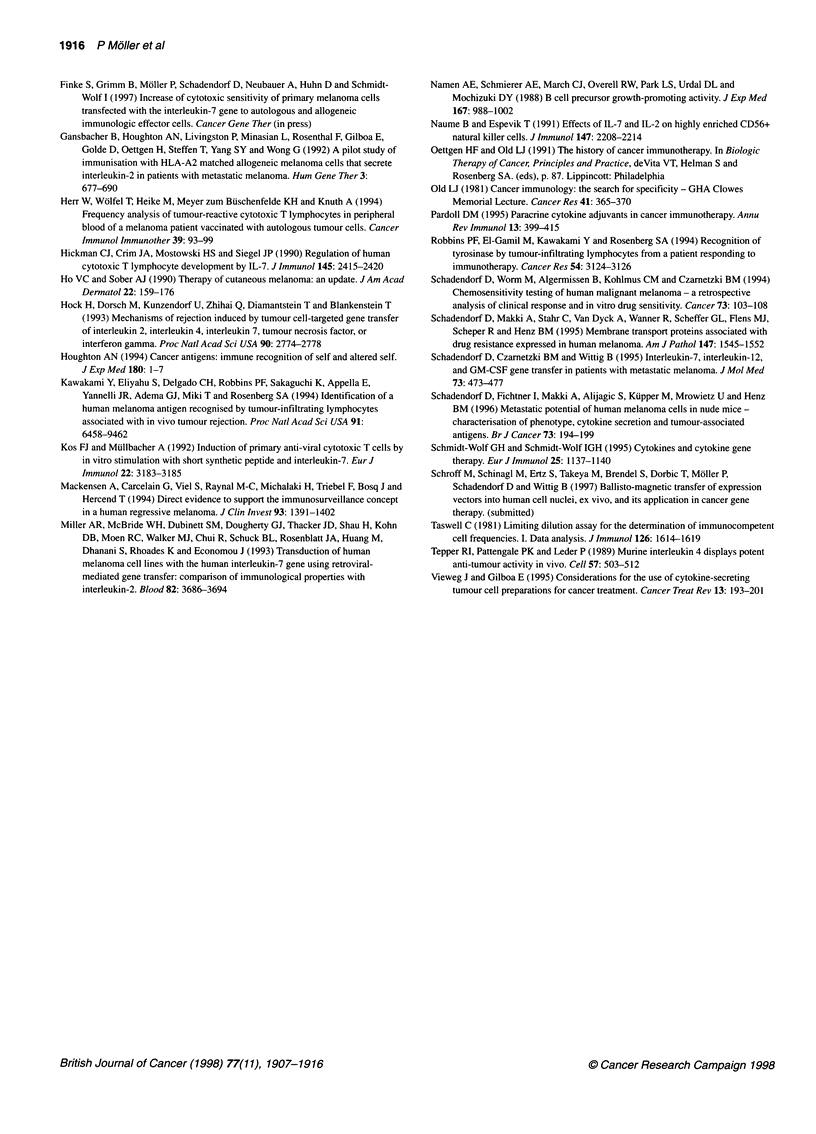# Vaccination with IL-7 gene-modified autologous melanoma cells can enhance the anti-melanoma lytic activity in peripheral blood of patients with a good clinical performance status: a clinical phase I study.

**DOI:** 10.1038/bjc.1998.317

**Published:** 1998-06

**Authors:** P. MÃ¶ller, Y. Sun, T. Dorbic, S. Alijagic, A. Makki, K. Jurgovsky, M. Schroff, B. M. Henz, B. Wittig, D. Schadendorf

**Affiliations:** UniversitÃ¤tshautklinik, Virchow Klinikum, Humboldt-UniversitÃ¤t zu Berlin, Germany.

## Abstract

Recently, cytokine gene transfer into tumour cells has been shown to mediate tumour regression in animal models via immunomodulation. Consequently, a number of clinical protocols have been developed to treat cancer patients with cytokine gene-modified tumour cells. Here, we report the results of a clinical phase I trial using for the first time autologous, interleukin 7 gene-modified tumour cells for vaccination of ten patients with disseminated malignant melanoma. Melanoma cells were expanded in vitro from surgically removed metastases, transduced by a ballistic gene transfer technique and were then injected after in vitro irradiation s.c. at weekly intervals. Clinically, there was no major toxicity except for mild fever, and no major clinical response towards vaccination was observed. Eight of ten patients completed the initial three s.c. vaccinations and were eligible for immunological evaluation. Post vaccination, peripheral mononuclear cells (PBMCs) were found to contain an increased number of tumour-reactive proliferative as well as cytolytic cells, as determined by a limiting dilution analysis. In three of six patients, the frequencies of anti-melanoma cytolytic precursor cells increased between 2.6- and 28-fold. Two of these patients showed a minor clinical response. Analysis of the autologous tumour cell vaccines regarding IL-7 secretion after gene transfer, HLA class I and class II cell surface expression, secretion of immunosuppressive mediators (TGF-beta1, IL-10) and various melanoma-associated tumour antigens revealed a very diverse expression profile. In conclusion, vaccination using gene-modified autologous melanoma cells induced immunological changes in a group of advanced, terminally ill patients. These changes can be interpreted as an increased anti-tumour immune response. However, immunological modulation was most pronounced in patients in good physical condition. Therefore, patients with minimal tumour load or minimal residual disease might preferentially benefit from tumour cell vaccination in further studies. In order to evaluate the effects of the cytokine gene-modified tumour cell vaccines more precisely, an antigenically better defined vaccine is needed.


British Journal of Cancer (1998) 77(11), 1907–1916
© 1998 Cancer Research Campaign

### Vaccination with IL-7 gene-modified autologous melanoma cells can enhance the anti-melanoma lytic activity in peripheral blood of patients with a good clinical performance status: a clinical phase I study

**P Möller[1,2,3], Y Sun[1,2,3], T Dorbic[2,4], S Alijagic[1,3], A Makki[1], K Jurgovsky[1,4], M Schroff[2,4], B M Henz[1], B Wittig[2,4] and D Schadendorf[1,2,3]**

[1]Universitätshautklinik, Virchow Klinikum, Humboldt-Universität zu Berlin, Berlin, Germany; [2]Centrum für Somatische Gentherapie e.V., Freie Universität, Berlin, Germany; [3]Clinical Cooperation Unit for Dermatooncology, (DKFZ), Theodor Kutzer Ufer 1, 68135 Mannheim, Germany; [4]Institute für Molekularbiologie und Bioinformatik, Freie Universität, Berlin, Germany

**Summary** Recently, cytokine gene transfer into tumour cells has been shown to mediate tumour regression in animal models via immunomodulation. Consequently, a number of clinical protocols have been developed to treat cancer patients with cytokine gene-modified tumour cells. Here, we report the results of a clinical phase I trial using for the first time autologous, interleukin 7 gene-modified tumour cells for vaccination of ten patients with disseminated malignant melanoma. Melanoma cells were expanded in vitro from surgically removed metastases, transduced by a ballistic gene transfer technique and were then injected after in vitro irradiation s.c. at weekly intervals. Clinically, there was no major toxicity except for mild fever, and no major clinical response towards vaccination was observed. Eight of ten patients completed the initial three s.c. vaccinations and were eligible for immunological evaluation. Post vaccination, peripheral mononuclear cells (PBMCs) were found to contain an increased number of tumour-reactive proliferative as well as cytolytic cells, as determined by a limiting dilution analysis. In three of six patients, the frequencies of anti-melanoma cytolytic precursor cells increased between 2.6- and 28-fold. Two of these patients showed a minor clinical response. Analysis of the autologous tumour cell vaccines regarding IL-7 secretion after gene transfer, HLA class I and class II cell surface expression, secretion of immunosuppressive mediators (TGF-β1, IL-10) and various melanoma-associated tumour antigens revealed a very diverse expression profile. In conclusion, vaccination using gene-modified autologous melanoma cells induced immunological changes in a group of advanced, terminally ill patients. These changes can be interpreted as an increased anti-tumour immune response. However, immunological modulation was most pronounced in patients in good physical condition. Therefore, patients with minimal tumour load or minimal residual disease might preferentially benefit from tumour cell vaccination in further studies. In order to evaluate the effects of the cytokine gene-modified tumour cell vaccines more precisely, an antigenically better defined vaccine is needed.

**Keywords:** gene transfer; cytolytic T lymphocyte; ballistic particle transfer; limited dilution transfer

Incidence of malignant melanoma has increased dramatically world-wide over the last few decades. Standard forms of cancer therapy, such as surgery, radiation and chemotherapy, although effective in certain types of cancer, fail to cure affected patients. In its advanced stage, melanoma has a high mortality because of its high resistance to conventional therapies (Ahmann et al, 1989; Ho and Sober, 1990; Schadendorf et al, 1994, 1995).

A number of clinical observations in human malignant melanoma underline the potential importance of the immune response in this disease (Old, 1981; Oettgen and Old, 1991; Ferrone, 1994; Mackensen et al, 1994). It is believed that the immune attack of infiltrating lymphocytes against melanoma cells may be responsible for the occurrence of spontaneous, partial or complete melanoma regression and for the concomitant destruction of melanocytes in benign lesions, leading to clinical phenomena, such as halo nevi, uveitis and vitiligo in melanoma patients. These observations, together with anecdotal and clinical reports of tumour regression after bacterial infections, application of bacterial vaccines or more specific immune interventions, suggest that melanoma is a good model for the evaluation of the various strategies of immune therapy (Old, 1981; Oettgen and Old, 1991; Ferrone, 1994; Mackensen et al, 1994). Recently, cytolytic T lymphocytes (CTL), which recognize and destroy tumour cells, have been isolated from blood- or from tumour-infiltrating lymphocytes of melanoma patients by numerous investigators (reviewed by Boon et al, 1994; Houghton, 1994). Furthermore, it has been demonstrated that such T lymphocytes are capable of mediating impressive tumour regression in vivo (Kawakami et al, 1994; Robbins et al, 1994).

The pioneering work of Tepper and co-workers (1989) showed that tumour cells were rejected after transfection of the IL-4 gene in an animal tumour model. In addition, Fearon and colleagues (1990) demonstrated that cytokines, after gene transfer, can bypass some of the co-stimulatory signals needed for T-cell priming so as to cause tumour rejection. Consequently, applications of numerous cytokine genes, including IL-7, have been tested in such animal models for their capacity to inhibit tumour growth and metastasis formation (reviewed by Horsch et al, 1993; Colombo and Forni, 1994; Vieweg and Gilboa, 1995).

*Received 23 April 1997*
*Accepted 3 November 1997*

*Correspondence to:* D Schadendorf, Clinical Cooperation Unit for Dermatooncology, (DKFZ), Theodor Kutzer Ufer 1, 68135 Mannheim, Germany

**Table 1** Patient characteristics and DTH reactivity [autologous melanoma cells and Multitest Merieux skin test (sum of total diameter given in mm/no. of reactive antigens)] before and after third vaccination

| Patient | | | | | | DTH reactivity (Multitest Merieux) | | DTH reactivity (autologous tumour cells) | |
|---|---|---|---|---|---|---|---|---|---|
| No. | Age/ sex | Sites of metastases | Previous therapies | HLA type | Karnofsky index | Before gene therapy | After third vaccination | Before gene therapy | After third vaccination |
| 1 | 51F | LN, lungs, skin, brain | Surgery, IFN + IL-2, DTIC + IFN | A1, A2, B60, B62, Cw3, DR4, DR13 | >70 | 13.5/3 | 11.5/3 | ∅ | ∅ |
| 2 | 63F | LN, skin, lungs, liver | Limb perfusion, DTIC, surgery | A11, A32, B7, B52, Cw5, DR1, DR2 | >70 | 21.5/3 | 16/4 | ∅ | ∅ |
| 3 | 52M | LN, liver, lungs | Vindesine + IFN, radiation | A2, B44, B51, Cw5, Cw8, DR4, DR13 | <70 | 2.5/1 | 6.0/2 | ∅ | ∅ |
| 4 | 29M | LN, skin, lungs | Surgery, radiation | A2, A32, B7, B60, Cw3, Cw7, DR4 | >70 | 32/4 | NA | ∅ | NA |
| 5 | 83M | LN, skin, liver, spleen, brain | Surgery, DTIC + IFN | A3, A19, B15, B39, Cw3, Cw7, DR7, DR9 | >70 | 4.0/1 | 6.0/2 | ∅ | ∅ |
| 6 | 26M | Skin, lungs, bone, LN | DTIC + DDP + BCNU IFN + IL-2, radiation | ND | <70 | 0.0/0 | NA | ∅ | NA |
| 7 | 33F | LN, skin, liver, lung, gut | Vindesine + IFN, IL-2, DTIC, DDP | ND | <70 | 0.0/0 | 0.0/0 | ∅ | ∅ |
| 8 | 48F | LN, lungs | Surgery, IL-2 + IFN, DTIC | A2, A11, B22, B75, Cw1, Cw3, DR1, DR15 | >70 | 18/3 | 3.0/1 | ∅ | ∅ |
| 9 | 52F | Skin, LN, | IFN + IL-2, DTIC, Fotemustine | A2, A24, B8, B62, Cw4 | <70 | 3/1 | 0.0/0 | ∅ | ∅ |
| 10 | 52M | Skin, LN, lungs, brain | Vindesine + IFN, DTIC, DDP, | A2, A9, B44, Cw4, DR7, DR11 | >70 | 13/2 | 8.0/3 | ∅ | ∅ |

ND, not done; NA, not applicable; ∅, no DTH reactivity; LN, lymph node.

IL-7 was originally identified as a factor required for the proliferation and maturation of pre-B cells, and this cytokine has subsequently been found to augment the growth and cytotoxicity of T cells (Hickman, 1990). In vitro, IL-7 is used to support APC-induced T-cell priming with a higher specificity than in the presence of IL-2 (Kos and Müllbacher, 1992; Celis et al, 1994). Compared with other cytokines, anti-tumour response induced by paracrine-secreted IL-7 seems to be more strictly dependent on T cells (Hock et al, 1993; Miller et al, 1993), although IL-7 is able to induce LAK activity (Alderson et al, 1990; Böhm et al, 1994), to enhance NK activity (24) and to generate tumoricidal activity in monocytes (Alderson et al, 1991). In a comparative study in a mouse tumour model using a murine plasmocytoma cell line, the IL-7-secreting gene-modified tumour cells showed, in contrast to other cytokine-secreting tumour cells, a dependency of CD4+ T cells on early tumour rejection, whereas CD8+ T cells were required for long-term tumour eradication (Hock et al, 1993).

Based on the successful animal studies using gene-modified tumour cells, various clinical protocols for the treatment of human cancer, predominantly using cytokine gene modifications, have been initiated in recent years. Indeed, only three reports on clinical gene therapy trials dealing with non-haematological neoplasms have been published as yet (reviewed by Culver, 1996).

A vaccination therapy using gene-modified autologous tumour cells is especially feasible in melanoma and is thought to present potentially all individual tumour antigens to the host immune system. To investigate the autologous vaccination approach, we manipulated autologous melanoma cells to overexpress IL-7 using a mammalian expression plasmid vector system and a ballistomagnetic gene transfer technique (Schadendorf et al, 1995*b*). A first clinical phase I trial was carried out to evaluate the feasibility and the clinical toxicity of such an approach. Furthermore, we report here for the first time on the modulation of immune response in a subset of patients with far advanced, metastatic melanoma after immunization with an IL-7 gene-modified, autologous tumour cell vaccine.

### MATERIALS AND METHODS

### Patient selection

According to the protocol published previously (Schadendorf et al, 1995*b*), accessible melanoma metastases were removed surgically, and melanoma cells were expanded in vitro (as described below) and transduced by ballistic gene transfer (see below). After magnetic enrichment of IL-7 gene-modified tumour cells and, after irradiation, patients were immunized s.c. at weekly intervals using multiple aliquots of the cell preparations. Patients were required to have histologically proven metastatic melanoma, adequate hepatic and renal function (bilirubin < 50 $\mu mol\ l^{-1}$; serum creatinine < 266 $\mu mol\ l^{-1}$) and needed to have at least one prior unsuccessful systemic treatment including chemotherapy, immunomodulators or a combination of both. A life expectancy of more than 8 weeks was required. Furthermore, patients with any severe cardiac or psychiatric disease as well as concurrent acute infection with hepatitis virus or HIV were excluded. All participants gave informed consent before enrolling in the study, as

© Cancer Research Campaign 1998

**Table 2** Cytokine gene transfer for treatment of malignant melanoma: scheme of immunological and clinical investigations

| | Number of days after gene transfer | | | | | | | |
|---|---|---|---|---|---|---|---|---|
| **Procedure** | **–7–0** | **1** | **8** | **15** | **29** | **36** | **50** | **64** |
| *Vaccination*[a] | | | | | | | | |
| Autologous melanoma cells plus gene encoding interleukin 7 | | + | + | + | | + | | + |
| *Venepuncture* | | | | | | | | |
| Blood samples for analysis of: lymphocyte proliferation; natural killer and lymphokine-activated killer cell activity; fluorescence-activated cell-sorting analysis; and cytotoxic T-cell precursor analysis | + | | | | + | | + | |
| *Immunological tests* | | | | | | | | |
| Delayed-type hypersensitivity skin tests for common recall antigens and autologous melanoma cells (irradiated) | + | | | | + | | + | |
| *Skin biopsies*[b] | | | | | | | | |
| Biopsies tested for presence of melanoma cells, lymphocytic infiltration and other irradiated cells | + | | | | + | | | + |
| *Clinical evaluation* | | | | | | | | |
| Chest radiography, ultrasound (lymph nodes, abdomen) and computerized tomography (brain) | + | | | | + | | | + |

[a]Vaccination can be continued at 4-week intervals, depending upon clinical response. [b]Skin biopsy is only made if a clinical response has been observed.

required by the Virchow Clinic Institutional Ethical Review Board and according to the Declaration of Helsinki. Treatment was carried out at the Department of Dermatology, Virchow Clinic, Berlin. Patient recruitment started in December 1994, and the study was closed in March 1996. Ten patients were enrolled and basic data are summarized in Table 1.

### Treatment

Within 1 week before vaccination, medical history was taken, and the following baseline studies were performed: a physical examination, haematological testing (haemoglobin, haematocrit, leucocyte and platelet count), blood chemistry panel and urinalysis. Blood was also taken for immunological testing. Delayed-type hypersensitivity skin tests were performed with the commercially available Multitest Merieux test, with cell lysates from autologous melanoma cells and with PBMCs before vaccination and at 5-week intervals thereafter. Chest radiography and computerized topographic scans of brain, chest and abdomen were performed unless previously obtained within 8 weeks. Eligible patients received three vaccinations at weekly intervals as shown in Table 2, according to the published protocol (Schadendorf et al, 1995*b*). In most instances, the vaccine preparation was split into equal aliquots of about $10^6$ cells each and administered s.c. intradermally in close proximity to the regional lymph nodes of each extremity. Comprehensive immunological screening, haematological testing and a crude clinical assessment (physical examination, chest radiography, ultrasound examination of the abdomen) was carried out before the fourth vaccination at day 36. A complete clinical and immunological screening similar to the initial work-up was carried out during treatment week 10 (day 64) for final evaluation. Patients were followed up until death.

### Clinical response and toxicity criteria

Although a clinical response could not be expected and was not the primary aim of this phase I trial, tumour sites were evaluated by physical examinations and scans at 6-week intervals. Standard definitions of major (complete or partial) objective responses were used (Schadendorf et al, 1994). A minor response (MR) was defined as a 25–50% decrease of lesion lasting at least 1 month, or a more than 50% decrease of lesions lasting less than a month. Stable disease (SD) was defined as less than a 25% change in size with no new lesions developing for 6 weeks. Survival was measured from diagnosis of first distant metastasis or start of vaccination using gene-modified autologous tumour cells. Adverse effects were recorded using common WHO toxicity criteria.

### Preparation of autologous melanoma cells

Tumour specimens were collected from patients with advanced melanoma undergoing procedures either as a part of the diagnostic work-up or for palliative treatment of their disease. Solid tumour specimens from lymph nodes, cut is or subcutis were placed immediately after removal into RPMI 1640 (Gibco, Eggenstein, Germany). Adjunct non-melanoma-containing tissue was removed as completely as possible by scalpel or scissors, and tumours were subsequently cut into pieces. After passing the pieces through a steel mesh with a pore size of 25 μm, the cells were washed twice and cultured in complete RPMI 1640 medium supplemented with 20% fetal calf serum (FCS; Seromed, Berlin, Germany). For vaccination, passages 2–10 were used. On histological and immunocytochemical examinations of cytospin preparations of these cultured cells, they were confirmed as being melanoma cells by a S-100 staining index of > 95%.

© Cancer Research Campaign 1998

### Polymerase chain reaction (PCR)

PCR was carried out with reversely transcribed cDNA generated from all melanoma cell lines as described previously (Schadendorf et al, 1996). Briefly, the following primer sequences were used: Tyr-1, TTG GCA GAT TGT CTG TAG CC and Tyr-2, AGG CAT TGT GCA TGC TGC TT, which generate a 284-bp DNA amplificate specific for tyrosinase; Tyr-3, GTC TTT ATG CAA TGG AAC GC and Tyr-4, GCT ATC CCA GTA AGT GGA CT, which generate a second 207-bp DNA amplificate specific for tyrosinase; MAGE1-3, CTT GCC TCC TCA CAG AG and MAGE1-5, TTG CCG AAG ATC TCA GGA A, which generate a 407-bp DNA amplificate specific for the MAGE-1 gene; MAGE3-5, TGG AGG ACC AGA GGC CCC C and MAGE3-3, GGA CGA TTA TCA GGA GGC CTG C, which generate a 714-bp DNA amplificate specific for the MAGE-3 gene; pMEL175, AGA TCC TGC AGG CTG TGC and pMEL173, CAA TGG GAC AAG AGC AGA, which generate a 540-bp DNA amplificate specific for the gp100/pMEL17 gene; MART1-5, ACT GCT CAT CGG CTG TTG and MART1-3, TCA GCC ATG TCT CAG GTG, which generate a 265-bp DNA amplificate specific for the MART-1/Melan-A gene.

### Determination of IL-10 and TGF-β1

Immunosuppressive mediators, such as IL-10 (Immunotech, Hamburg, Germany) and TGF-β1 (Genzyme, Cambridge, MA, USA), released by melanoma cells used for vaccination were performed by quantitative immunoenzymometric kits. Then, $2 \times 10^5$ melanoma cells in 2 ml of complete medium were seeded in 24-well plates at 37°C in a 5% carbon dioxide atmosphere for 24 h. Cytokine levels were determined in the supernatants. The sensitivity was 0.05 ng $ml^{-1}$ for TGF-β1 and 5 pg $ml^{-1}$ for IL-10, respectively, as indicated by the manufacturers.

### Maintenance of cell cultures

Newly established human melanoma cell lines of patients were grown in complete medium (RPMI 1640 supplemented with 10% FCS, 2 mM glutamine (Gibco) and 100 U $ml^{-1}$ penicillin/streptomycin (Seromed). Cultures were maintained at 37°C in an atmosphere of 5% carbon dioxide in air. The NK-sensitive cell line K562, autologous EBV-immortalized B cell lines and autologous melanoma cells, all grown in complete medium, were used as further target cells in the immunological assays. The T-cell lines were generated and maintained in T-cell medium (RPMI containing 10% pooled human AB serum (Sigma, Deisenhofen, Germany), 25 mM HEPES buffer, 2 mM glutamine, 100 U $ml^{-1}$ penicillin, 100 μg/$ml^{-1}$ streptomycin). After 4 weeks of limited dilution (LD) culture, growth of generated T cell lines was further supported by addition of autologous inactivated EBV-immortalized B cell lines. For the LDH release assay, a separate medium consisting of RPMI 1640 without phenol red (Seromed, Berlin, Germany) was used supplemented with 2 mM glutamine, 100 U $ml^{-1}$ penicillin, 100 μg $ml^{-1}$ streptomycin and 3% FCS (medium B).

### Ballistomagnetic gene transfer for expression of plasmids into melanoma cells

The full-length human IL-7 gene was cloned into a eukaryotic expression vector pRc/CMV (Invitrogen, Heidelberg, Germany) and entirely sequenced before use as described (Schadendorf et al, 1995*b*). For gene transfer, the newly developed ballistomagnetic vector system, which can efficiently transfer nucleic acids and other biomolecules into the nuclei, up to $1 \times 10^8$ cells were used, which allowed us to obtain >90% of pure transfected cells in less than 1 h. Briefly, the ballistomagnetic vector system is a two-step procedure, i.e. the actual transfer of nucleic acids together with superparamagnetic particles into the nuclei of many cells, followed by a magnetic isolation of transfected cells. At the first step, a suspension of colloidal gold (0.9 mg, 1.6 ptm, BioRad; Germany) was pipetted onto each of seven particle carrier membranes (purchased as macro-carrier from BioRad) and allowed to sediment. After removal of the supernatant, the gold particles were resuspended in a mixture of three parts of an aqueous solution of DNA and two parts of a suspension of colloidal superparamagnetic particles of 30-nm diameter (Miltenyi, used as purchased) and were allowed to dry onto the particle carrier membranes at room temperature. The accelerating system for ballistic transfer is based on the biolistic PDS-1000/He apparatus (1550 psi rupture disk, 20 inches Hg of vacuum, BioRad). After ballistomagnetic transfer, cells were immediately resuspended in 5 ml of phosphate-buffered saline (PBS) and transferred onto a high-gradient magnetic separation column (capacity $3 \times 10^7$ cells, type AS, Miltenyi), which was prepared according to the supplier's protocol. An aliquot of the cell suspension was kept for reference (unsorted fraction). The cell suspension was passed through the column, followed by a washing step with 3 ml of PBS. After removal of the column from the magnetic separator, the retained cells were flushed back to the top of the column. Finally, the column was removed from the separator and eluted with 5 ml of PBS (magnetic fraction). Recovered cells were sedimented at 400 ***g*** at 4°C for 7 min and resuspended in tissue culture medium. Cells were incubated under culture conditions for 24 h.

### IL-7 secretion after gene transfer and irradiation

The following day, gene-modified melanoma cells were irradiated with 100 Gray in a small volume. Cells were subsequently detached from flasks by mechanical scraping and ice-cold PBS, washed three times with sterile PBS and finally counted and resuspended with $5 \times 10^6$ gene-modified melanoma cells per ml. An aliquot of $10^6$ cells was transferred to a single well of a 24-well plate (Nunc) for determining cytokine secretion after 24 h, using an IL-7 ELISA (EIAKIT PerSeptive Diagnostics, Cambridge, MA, USA). Detection range was between 10 and 200 pg $ml^{-1}$ according to the manufacturer's instructions. The biological activity of the IL-7-containing supernatant was analysed and confirmed with a bioassay that used the IL-7-dependent IxN/2b pre-B-cell line as described (Namen et al, 1988).

### Immunological studies

### *Delayed-type hypersensitivity (DTH)*

DTH tests were performed 2 days before the first vaccination and during weeks 6 and 12 of treatment. Two preparations were used each time, one consisting of an autologous melanoma cell lysate and the other consisted of autologous peripheral blood mononuclear cell (PBMC) lysate as a negative control. The melanoma test preparation was prepared from autologous cells of each patient, starting with $4 \times 10^6$ suspended, mitomycin-inactivated (45 min, 37°C)

 © Cancer Research Campaign 1998

primary melanoma cells. After PBS washings, cells were lysed by three cycles of freeze–thawing. Aliquots of $1.3 \times 10^6$ cell equivalents were stored in 0.3 ml of PBS per tube at –80°C. The autologous PBMC lysate was prepared after separation of leucocytes by ficoll centrifugation using the same procedures as described above for melanoma cells. For DTH testing, $1 \times 10^6$ cell equivalents were injected intradermally into the forearm. In parallel, a commercially available recall DTH test (Multitest Merieux), was administered on the opposite forearm. A positive skin test reaction was defined as >5 mm diameter induration after 48 h.

### *Preparation of autologous lymphocytes*

PBMCs were isolated from heparinized venous blood of patients by Ficoll-Hypaque (Biorad, Berlin, Germany) density centrifugation, washed two times with PBS and either resuspended in complete culture medium (see target cells) or cryopreserved in liquid nitrogen in the presence of more than 50% FCS. EBV-immortalized B cell lines were used as autologous targets and as feeder cells supporting T-cell growth. Immortalized B cell lines were generated using a standard method (Blumberg et al, 1987) using B95-8 marmoset cell line supernatant containing EBV.

### *Limiting dilution microcultures*

To estimate changes in the frequency of tumour-reactive T cells as well as tumour-specific cytolytic T-lymphocyte (CTL) precursors in the peripheral blood, we used a limiting dilution analysis (LDA) method, described by Coulie et al (1992) with minor modifications. Briefly, cryopreserved PBMCs obtained before vaccination and 2 weeks after the third vaccination were seeded at limiting dilutions in microcultures of 96 V-bottom microwells (Nunc, Roskilde; Denmark) with $1 \times 10^4$ mitomycin-inactivated autologous melanoma cells in the presence of 20 U $ml^{-1}$ IL-2 and 200 µl of medium. Cell viability was required to be above 85% by trypan blue exclusion, and cell counts were performed by two independent investigators (PM, YS). At least 48 microcultures were set up for each dilution of PBMCs (10 000, 5000, 2500, 1250, 625, 312 cells per well). On day 14, the cells were transferred into flat-bottom microwells (Nunc). Microcultures were restimulated on days 7, 14 and 21 with $1 \times 10^4$ inactivated melanoma cells per well with 20 U $ml^{-1}$ IL-2 in either 100 µl of fresh medium/V-bottom well or 200 µl of fresh medium/flat-bottom well. Cultures were washed once and resuspended in 200 µl of medium B. Three aliquots of 60 µl were transferred into U-bottom microwells (Nunc) to test their lytic activity in a LDH release assay.

### *Determination of proliferative T-cell response*

Proliferative T-cell response could be assessed only by microscopic inspection because of the limited number of cells needed for cytolytic assessment. Therefore, all LDA microcultures were scored semiquantitatively at day 25 for proliferative response. The following criteria to identify visible clones in LDA microcultures were used: ∅, no cell clusters, less than 15% of bottom covered by proliferating cells; +++, bottom of well covered more than 50% with proliferating PMBC-derived cells; ++, bottom of well covered between 25% and 50%; +, bottom of well covered between 15% and 25% by proliferating blood cells.

### *Determination of cytotoxicity of responder lymphocytes upon co-culture with autologous melanoma cells using the LDH release assay*

All microculture wells were analysed regarding cytolytic activity using the LDH release assay. The LDH release assay is a colorimetric enzyme release test that showed a good correlation to the radioactive $^{51}Cr$ release assay (Decker et al, 1988). Here, it was used to measure the lytic activity generated in the LDA microcultures against non-adherent target cells (e.g. the NK-sensitive cell line K562) and autologous melanoma cells. For further T-cell line characterization, lyric activity was also tested against autologous EBV-immortalized B cells.

The assay was performed using a commercially available detection kit for LDH (Boehringer Mannheim, Mannheim, Germany) that detects released LDH by reduction of a tetrazolium salt (INT) to a water-soluble red formazan salt mediated by NAD+/NADH and lactate/pyruvate. Briefly, effector cells obtained from LDA

**Table 3** Number of gene-modified cells used for each vaccination administered into multiple injection sites (number given in parentheses). IL-7 release of 'vaccination batch' of cells after irradiation (100 Gy) given as pg of IL-7 $ml^{-1}$ $10^{-6}$ cells $day^{-1}$. Total number of gene-modified melanoma cells and total IL-7 dose levels given were calculated after third vaccination

| Patient | | Number and IL-7 release of cells used for vaccination | | | | | | |
|---|---|---|---|---|---|---|---|---|
| Number | First dose | IL-7 release 'first dose' | Second dose | IL-7 release 'second dose' | Third dose | IL-7 release 'third dose' | Total number of cells administered | Total IL-7 dose received |
| 1 | $1 \times 10^7$ (3) | 1031 | $1 \times 10^7$ (4) | 746 | $1 \times 10^7$ (4) | 927 | $3 \times 10^7$ | 27 040 |
| 2 | $1 \times 10^6$ (2) | 713 | $2 \times 10^6$ (2) | 310 | $3 \times 10^6$ (2) | 202 | $6 \times 10^6$ | 1939 |
| 3 | $2 \times 10^6$ (2) | 511 | $2 \times 10^6$ (3) | 701 | $1 \times 10^6$ (2) | 668 | $5 \times 10^6$ | 3092 |
| 4 | $2 \times 10^6$ (2) | 231 | $4 \times 10^6$ (3) | 182 | Not given | NA | $6 \times 10^6$ | 1190 |
| 5 | $2 \times 10^6$ (2) | 406 | $1.5 \times 10^7$ (4) | 424 | $6 \times 10^6$ (4) | 402 | $2.3 \times 10^7$ | 9584 |
| 6 | $5 \times 10^5$ (2) | ND | Not given | NA | Not given | NA | NA | NA |
| 7 | $1 \times 10^6$ (2) | 900 | $1 \times 10^6$ (2) | 1162 | $1 \times 10^6$ (2) | 1560 | $3 \times 10^6$ | 3622 |
| 8 | $3.2 \times 10^6$ (3) | 885 | $1 \times 10^6$ (2) | 1131 | $1.1 \times 10^7$ (4) | 485 | $1.5 \times 10^7$ | 8814 |
| 9 | $5 \times 10^5$ (1) | 1170 | $2 \times 10^6$ (2) | 604 | $3 \times 10^6$ (3) | 490 | $5.5 \times 10^6$ | 3263 |
| 10 | $4 \times 10^6$ (4) | 515 | $2.7 \times 10^6$ (3) | 380 | $4 \times 10^6$ (4) | 635 | $1.1 \times 10^7$ | 5628 |

ND, not done; NA, not applicable.

© Cancer Research Campaign 1998

**Table 4** Characterization of melanoma cell vaccines used for the immunization

| Patient no. | Patients' surface markers (MFI) | | | | TGF-β1 release | Expression of tumour antigens (RT-PCR) | | | | | |
|---|---|---|---|---|---|---|---|---|---|---|---|
| | HLA-A,B,C | HLA-A2 | HLA-DR | HLA-DQ | (ng ml$^{-1}$ 10$^{-5}$ cells day$^{-1}$) | Tyr-1/2 | Tyr-3/4 | gp100 | MART-1 | MAGE-1 | MAGE-3 |
| 1 | 64.5 | 1.1 | 1.6 | 1.5 | 0.33 | +/– | + | + | + | – | – |
| 2 | 126.4 | ND | 70.4 | 3.6 | 0.47 | + | + | + | +/– | – | + |
| 3 | 71.1 | 10.4 | 1.0 | 1.0 | 0.85 | ND | | | | | |
| 7 | 158.2 | ND | 274.5 | 1.2 | 0.41 | + | + | – | – | + | +/– |
| 8 | 67.9 | 9.4 | 1.0 | 1.0 | 0.37 | + | + | + | +/– | – | – |
| 9 | 44.6 | 4.4 | 1.0 | 1.0 | 0.74 | – | – | +/– | – | – | – |
| 10 | 46.9 | 3.7 | 5.1 | 1.1 | 0.66 | + | + | + | +/– | – | + |

MFI (mean fluorescence index) = fluorescence of specific stained cells/fluorescence of negative control staining; ND, not done; –, not detectable; +, specific amplification; +/–, questionable, at the detection level.

microcultures were washed and split into three aliquots of 60 µl and transferred to 96-well U-bottom microplates (Nunc). Then, $5 \times 10^3$ target cells (autologous melanoma or K562 cells) in 100 µl per well medium B were added (designed as 'A experimental'). Plates were incubated for 6 h at 37°C in 5% humified carbon dioxide. After centrifugation at 250 ***g*** for 7 min, supernatants were transferred to corresponding wells of 96 flat-bottom microwells (Nunc), and 100 µl of test kit solution (catalyst and dye solution) was added to each well. After incubation for 30 min at room temperature, the absorbance (A) at 492 nm wavelength was determined using an ELISA reader (Titertek Multiscan MCC/340, Meckenheim, Germany). Medium B served as background control. The specific lysis was calculated according to the formula:

$$\text{Lysis (\%)} = \frac{\text{(A experimental – A effector cell spontaneous) – A target cell spontaneous}}{\text{A target cell total – A target cell spontaneous}} \times 100$$

'A effector cell spontaneous' and 'A target cell spontaneous' were determined from the test solution, which contains a 60-µl aliquot of the LDA microculture without target cells plus medium B; 'A target cell total' (maximal release) was obtained after treatment with 1% triton X 100 (Sigma); 'A target cell spontaneous' ranged between 10% and 20% of total release.

### *Analysis of T-cell reactivity in microcultures*

All microcultures with an LDH release exceeding the mean spontaneous release from target cells (T) (measured in 12 control wells) by at least three standard deviations were considered to be cytolytically positive. The statistical method of precursor cell frequency, 95% confidence intervals and *P*-values indicative of single-hit kinetics were determined by a computer program based on published methods (Taswell, 1981) kindly provided by Dr Heeg (München, Germany).

### *Flow cytometry*

One-colour analysis was performed on an EPICS XL (Coulter, Krefeld, Germany) as described (Böhm et al, 1994). Aliquots of $4 \times 10^5$ trypsinized melanoma cells in PBS containing 0.1% sodium azide were incubated with antibodies for 30 min at 4°C. Staining of cell surface markers of melanoma cells was performed using unlabelled antibodies detecting HLA-A,-B,-C, HLA-DR, HLA-DQ, ICAM-1 (all obtained from Immunotech, Hamburg, Germany) and HLA-A2 (Clone BB7.2; a gift from Dr Coulie, Brussels, Belgium) and the respective isotype control antibodies in combination with a FITC-labelled goat anti-mouse Ig antibody (Immunotech).

### *Statistical analysis*

Statistical significance of the data obtained from the cytoxicity assays and IL-4 measurements were calculated using a SPSS computer package. A modified Wilcoxon signed-rank test and a Mann–Whitney *U*-test were used.

### RESULTS

### Clinical assessment of the course of disease

Ten patients (Table 1) were enrolled in the study. All patients suffered from advanced metastatic melanoma, with the mean disease-free interval from excision of the primary tumour to the first distant metastasis amounting to 24.5 months (not shown). As all patients had received various conventional oncological treatment regimens, an additional 9.6 months had elapsed on average before vaccination was started (not shown). Approximately 50% ($n = 19$) of melanoma metastases received could be sufficiently expanded in vitro. Vaccination treatment could be initiated between 2 weeks and 3 months after surgical removal of the metastases in ten patients with the remaining nine patients having died meanwhile. Patients received between $5 \times 10^5$ (minimal required number) and $1.5 \times 10^7$ autologous, IL-7 gene-modified melanoma cells per vaccination as shown in Table 3. IL-7 secretion of gene-modified cells varied between 182 and 1560 pg ml$^{-1}$ 10$^{-6}$ cells during 24 h, with the total number of gene-modified cells administered ranging between $3 \times 10^6$ and $3 \times 10^7$ and a calculated total IL-7 dose between 1.19 and 27.04 ng (Table 3). Preclinical studies demonstrated that after irradiation of gene-modified melanoma cells with 100 Gy, transfected cells increased IL-7 secretion for 1–3 days in vitro, and subsequently production of IL-7 was decreasingly detectable over 2 weeks (Finke et al, 1997). Eight of ten patients received the first three immunizations and were evaluable in week 5. One patient (no. 6, DB) died after the first vaccination as a result of disease progression and unrelated to the vaccination. A second patient (no. 4, EB) refused further immunization after the second injection (Table 3). No major clinical response (CR, PR) was observed in any patient. Four patients (no. 1, no. 2, no. 3, no. 5) showed stable disease and two a mixed response (no. 8, no. 10). These six patients received a fourth vaccination, but further immunization was terminated at the next hospital visit in all cases because of tumour progression.

© Cancer Research Campaign 1998

**Table 5** Frequency analysis of tumour-reactive lymphocytes in PBMCs

| Patient no. | Proliferative activity | | Total lytic activity[a] | |
|---|---|---|---|---|
| | B | A | B | A |
| 1 | 1/3987 | 1/3677 | 1/8959 | 1/8345 |
| 2 | 1/3458 | 1/3032 | 1/323 413 | 1/140 119 |
| 3 | 1/4671 | 1/3325 | 1/59 173 | 1/47 595 |
| 7 | 1/24 058 | 1/9883 | [b] | [b] |
| 8 | 1/8351 | 1/1452 | 1/135 065 | 1/5381 |
| 9 | [c] | [c] | [c] | [c] |
| 10 | 1/3970 | 1/3342 | 1/29 176 | 1/7114 |

[a]Lytic activity against autologous melanoma cells or K562. [b]No lytic cells were generated. [c]Did not follow single-hit kinetics with *P*-values > 0.1. B before vaccination; A after vaccination.

**Table 6** LD cultures with lytic activity against autologous melanoma cells[a] and frequency analysis (f) of anti-melanoma lytic cells in PBMCs

| Patient no. | B | f | A | f |
|---|---|---|---|---|
| 1 | 13 (4) | 1/59 758 | 25 (7) | 1/23 187 |
| 3 | 3 (0) | [b] | 2 (0) | [b] |
| 8 | 4 (3) | 1/159 700 | 79 (20) | 1/5740 (1/36 659) |
| 10 | 2 (0) | [b] | 10 (3) | [b] |

[a]Number of LD cultures with lytic activity either against autologous melanoma only or against autologous melanoma and K562 in one LD culture. In parentheses: LD cultures and frequency, respectively, with specific lytic activity (lytic activity against autologous melanoma cells only). [b]Did not follow single-hit kinetics, with *P*-values > 0.1. [f]Frequency in PBMCs

Patients died on average 4 months after initiation of vaccination with IL-7 gene-modified tumour cells, demonstrating their poor physical condition overall. Immunologically, responsive patients (no. 1, no. 8, no. 10) survived for 7.3 months on average (3, 10, 9 months respectively). Patient no. 8 showed an 8-month stabilization of disease with cutaneous metastases appearing and disappearing in short intervals.

### Immune status

### *Skin reactivity*

Delayed-type hypersensitivity (DTH), i.e. T-cell reactivity with common recall antigens, such as bacterial antigens, tetanus toxoid, etc., as determined by the Multitest Merieux and reflecting the overall immunological status of the patients, was already dramatically reduced in five patients (no. 3, no. 5, no. 6, no. 7, no. 9) before therapy. In addition, four of the six patients had a Karnofsky index below 70 (Table 1). No specific DTH reactivity using autologous melanoma cells was observed in any patient at any time point after intradermal injection of autologous melanoma cell or peripheral blood cell lysates (Table 1).

### *Characterization of vaccines*

To evaluate possible interactions between immune effecter cells and melanoma cells used as tumour vaccines, we characterized the autologous melanoma cells regarding the expression of cell surface markers (HLA-A,-B,-C, HLA-A2, HLA-DR, HLA-DQ and ICAM-1) using FACS, the release of immunosuppressive mediators (IL-10 and TGF-β1) using ELISA and tumour antigens known to be recognized by T cells (tyrosinase; gp100, MART-1, MAGE-1 and MAGE-3) using RT-PCR. All melanoma cells used for vaccination demonstrated high reactivity with MAb recognizing HLA-A,-B,-C (Table 4), as they did with ICAM-1 (not shown). Peripheral blood lymphocytes (PBls) from patients no. 1, no. 3, no. 8, no. 9 and no. 10 were typed HLA-A2 positive (Table 1), whereas patient no. 1 showed a loss of HLA-A2 expression on her melanoma cells that could not be up-regulated by IFN-γ (not shown). HLA-DR expression on melanoma cells was detected in two patients (no. 2, no. 7; Table 4). TGF-β1 secretion by tumour cells varied between 0.33 to 0.85 ng $ml^{-1}$ $10^{-5}$ cells $day^{-1}$ (Table 4), whereas IL-10 secretion was not detectable in all cell lines (not shown). Expression of tumour antigens was determined by RT-PCR and demonstrated the presence of tyrosinase (primers Tyr-1/2, Tyr-3/4) and gp100 in five of six melanoma lines analysed. MART-1/Melan-A was detected in one patient's tumour cells (no. 1) abundantly and in three others at the detection level. MAGE-1 was not expressed in cell lines tested, whereas MAGE-3 was detected in patients' no. 2, no. 7 and no. 10 melanoma cells. Interestingly, melanoma cells of patient no. 8, who demonstrated the highest increase of the anti-melanoma response in LD cultures after vaccination (Table 6), secreted only small amounts of TGF-β1, expressed strongly the HLA-A2 and three known tumour antigens known to be recognized by T cells in a HLA-A2-dependent fashion (Table 4).

### *Frequency analysis of tumour-reactive lymphocytes*

Our aim was to determine the changes of the precursor frequencies of tumour-reactive lymphocytes in PBMCs before and after the vaccinations. This could be realized in most patients by setting up limiting dilution (LD) microcultures to obtain mixed lymphocyte tumour cultures (MLTCs) in a statistically sufficient number and distribution for a mathematical evaluation. As a side-product, we generated several T cell lines at the end of the LD culture periods.

Quantitative results of lytic clones and semiquantitative results of proliferative clones from PBMCs obtained before the first and after the third vaccination could be determined in seven patients after co-culture with autologous melanoma cells over 25 days in vitro. Proliferative T-cell response could be determined only by microscopic inspection as the cell number was too low to perform thymidine incorporation and cytolytic assessment in parallel. Six patients were evaluable for frequency analysis of tumour-reactive proliferation and cytotoxicity (Table 5). Four out of six evaluable patients showed an increase in the frequency of proliferative and/or cytolytic precursor lymphocytes after vaccination. Two patients had a significant increase of tumour-reactive proliferation in responder lymphocytes (no. 7, 4.1-fold; no. 8, 5.75-fold; $P < 0.01$). An increase in lytic activity directed against autologous melanoma cells could be detected in three patients (Table 6; no. 1, no. 8, no. 10). In patients no. 1 and no. 8, the precursor frequency analysis of anti-melanoma lyric activity as well as of tumour-reactive, proliferative lymphocyte cultures could be calculated, and they demonstrated a 2.6-fold (no. 1) and a 28-fold (no. 8) increase of lytic activity against autologous melanoma cells in peripheral blood upon immunization (Figure 1). We could not perform a target cell competition with the NK-sensitive target cell

**Figure 1** Precursor analysis of proliferative and cytolytic anti-melanoma lytic lymphocytes. Increase in frequency of proliferative and cytolytic anti-melanoma lytic lymphocytes in PBMCs of patient no. 1 (**A** and **B**) and patient no. 8 (**C** and **D**). Limited dilution analysis was performed as described in Materials and methods with PBMCs obtained before (■) and two weeks after the third (□) vaccination by LDH test line K562 to abolish the NK-like activity as the LDH release assay was used for cytolytic testing. However, cytotoxicity against NK-sensitive cell line K562 and melanoma cells were analysed in parallel. By analysing LD cultures with lytic activity against autologous melanoma cells but without cytotoxicity against K562, an increase of the melanoma-specific cytotoxic T cell precursor (pCTL) frequency of more than fourfold for patient no. 8 was observed (Table 6).

Further analysis of 34 cytolytic T cell lines of three patients revealed that 32 out of 34 T cell lines exhibited specific reactivity against autologous melanoma cells without cross-reactivity against autologous EBV B cells. Preliminary analysis of seven out of seven CTL lines obtained from patients no. 1 and no. 8 demonstrated a HLA class I-dependent recognition of the melanoma cells, as could be shown by blocking experiments using the HLA class I-neutralizing MAb W6/32 (kindly provided by Dr P Coulie). Furthermore, in patient no. 1, recognition of tumour cells by T cells was HLA-B/HLA-C and not HLA-A2 restricted, as HLA-A2 was lost (Table 4), and HLA-A2-blocking antibody BB7.2 had no effect on T-cell-mediated cell lysis.

### Adverse effects and toxicity

Vaccinations were well tolerated by all patients without any signs of toxicity. No erythema, swelling or induration was detectable in any of the patients. Mild fever (grade I–II) and mild flu-like symptoms were observed in two patients (no. 2, UH, no. 10, LA) with temperatures up to 39°C, lasting up to 24 h post immunization.

### DISCUSSION

Previous animal studies indicate that a potent protective immune response can be generated in vivo using cytokine gene-modified tumour cells (reviewed by Colombo and Forni, 1994; Vieweg and Gilboa, 1995). The possible mechanisms by which cytokine-modified tumour cells may function as vaccines have been reviewed elsewhere (Finke et al, 1997). Active immunotherapy of certain human cancers, including malignant melanoma, using cytokine gene modification of autologous tumour cells is currently being tested in a number of clinical trials (reviewed by Pardoll, 1995). We report here on the results of the first clinical phase I trial using autologous, IL-7 gene-modified tumour cells for the treatment of ten patients with advanced metastatic melanoma. This pilot study demonstrates the feasibility and safety as well as the lack of toxicity of such an approach. Although five patients showed stable disease and two mixed response for some time, no major clinical response (CR, PR) was achieved.

However, in three of seven patients, immunological monitoring suggested an increase of anti-melanoma lytic clones (up to 28-fold) in the peripheral blood, comparing the pre- to post vaccination status. Frequency analysis of tumour-reactive lymphocytes has been used by others, with good results 2 weeks after vaccination (Schmidt-Wolf and Schmidt-Wolf, 1995). Whether or not the immunological changes observed are caused by the IL-7 transfection could not be tested in this initial phase I study. Ethical requirements did not allow a control group with non- or mock-transfected tumour cells at this point. Nevertheless, experiments using human

© Cancer Research Campaign 1998

melanoma cell lines transfected with the IL-7 gene demonstrated an advantage of transfected melanoma cells similar to non-transfected cells plus the addition of exogenous IL-7 for the generation of cytotoxic lymphocytes in vitro (Miller et al, 1993). Furthermore, proliferation and cytotoxicity of the T cells could be driven by paracrine IL-7 even in concentrations secreted by the administered vaccines (Finke et al, 1997). The exact dosage of IL-7 needed to mediate an anti-tumour effect in vivo is presently not clear. Hock et al (1991) used $4 \times 10^6$ tumour cells secreting around 20 ng $ml^{-1}$ for tumour transplantation onto mice, however this situation is not mimicking the human situation with a widely disseminated disease at the start of treatment. IL-7 has also been reported to be able to break tolerance of tolerized and anergic T cells (Filion et al, 1995). Nevertheless, no sign of inflammation has been observed at the vaccination sites in our clinical study.

In this clinical trial, immunologically responsive patients were characterized by a Karnofsky index above 70 and a marked response against recall antigens (Multitest Merieux) before vaccination. Furthermore, they received a large number of autologous melanoma cells ($> 10^7$ cells) secreting a high amount of IL-7 (> 5000 pg). Whether Karnofsky index, Multitest reactivity or cell number used for vaccination are relevant is difficult to assess because of the small number of patients treated. Interestingly, the melanoma cells from two of three immunologically responsive patients secreted only low amounts of the immunosuppressive cytokine TGF-$\beta$1 in a group of seven investigated patients. Preliminary characterization of cytolytic T cell lines generated after vaccination suggests that not the commonly known melanoma-associated antigens, such as MAGE-1, MAGE-3, tyrosinase, Melan-A or gp100, are recognized in the context of HLA-A2, but possibly so far unknown melanoma antigens presented by HLA-B or -C alleles.

In conclusion, vaccination with gene-modified tumour cells seems to be feasible and well tolerated. However, autologous tumour vaccines, which should have all relevant tumour antigens for the individual patient and pose no problems regarding HLA incompatibilities, are very labour, cost and time intensive to prepare. A major problem in the preparation of autologous tumour cell vaccines is the long latency period that was (sometimes) needed to expand the tumour cells in vitro; several of the patients substantially worsened in their clinical performance status. Furthermore, adjuvant immunization, which is more likely to alter the clinical course in tumour patients, is severely hampered by the requirement to obtain autologous tumour tissue. Therefore, besides using autologous tumour cells for gene transfer and vaccination, some clinical trials are under way using allogeneic tumour cell lines for vaccination (Gansbacher et al, 1992; Schmidt-Wolf and Schmidt-Wolf, 1995). This approach requires comparably less extensive cell preparations and allows for easier standardization, although the benefit of a strong allogeneic reaction in such an immunization approach is not sufficiently evaluated. Particularly, the loss of HLA alleles on the tumour cells, as observed in one of our patients (and two other patients not included in this study), and the need for a number of well-defined tumour antigens to be expressed favour, at this point, the allogeneic immunization. Vaccination trials using allogeneic, well-defined (regarding tumour antigens, secretion of immunosuppressive mediators and expression of HLA molecules) tumour cell vaccines that can use easier-to-standardize immunological detection assays for a number of defined tumour antigens should allow the evaluation of the potential and the limits of this new treatment modality in the near future.

### ACKNOWLEDGEMENTS

This work was supported by the DFG (Scha 422/3-2, Scha 422/5-1 and Scha 422/6-1) and by the Centrum Somatische Genetherapie e.V. DS is a Heisenberg-Fellow of the DFG. The authors are grateful to LJ Old (Ludwig Institute for Cancer Research, NY, USA) for providing the human melanoma cell lines. The active support of Professor Gollnick, Madgeburg, and the excellent technical assistance of Mrs Antje Sucker, Helga Kemmer and Iris Ziglowski are gratefully acknowledged.

### ABBREVIATIONS

IL, interleukin; LAK, lymphokine-activated killer; PBL, peripheral blood lymphocytes; MTT, 3-[4,5-dimethylthiazol-2-yl]-2,5-diphenyltetrazolium bromide; NK, natural killer; LDH, lactate dehydrogenase, DTH, delayed-type hypersensitivity; DMSO, dimethyl sulphoxide; PBS, phosphate-buffered saline; PBMCs, peripheral blood mononuclear cells; LDA, limiting dilution analysis; APC, antigen-presenting cells; FCS, fetal calf serum; CTL, cytolytic T lymphocyte

### REFERENCES

Ahmann DL, Creagan ET, Hahn RG, Edmonson JH, Bisel HF and Schaid DJ (1989) Complete responses and long-term survivals after systemic chemotherapy for patients with advanced malignant melanoma. *Cancer* **63**: 224–227

Alderson MR, Sassenfeld HM and Widmer RB (1990) Interleukin-7 enhances cytolytic T lymphocyte generation and induces lymphokine-activated killer cells from human peripheral blood. *J Exp Med* **172**: 577–587

Alderson MR, Tough TW, Ziegler SF and Grabstein KH (1991) Interleukin-7 induces cytokine secretion and tumoricidal activity by human peripheral blood monocytes. *J Exp Med* **173**: 923–930

Blumberg RS, Panadis T, Byington R, Henle W, Hirsch MS and Schooley RT (1987) Effects of human immunodeficiency virus on cellular immune response to Epstein–Barr virus in homosexual men: characterisation of cytotoxic response and lymphokine production. *J Infect Dis* **155**: 877–890

Böhm M, Möller P, Kalbfleisch U, Worm M, Czarnetzki BM and Schadendorf D (1994) Lysis of allogeneic and autologous melanoma cells by IL-7-induced lymphokine-activated killer cells. *Br J Cancer* **70**: 54–59

Boon T, Coulie P, Marchand M, Weynants P, Wölfel T and Brichard V (1994) Genes coding for tumour rejection antigens: perspectives for specific immunotherapy. In *Important Advances in Oncology*, DeVita VT, Hellman S and Rosenberg SA. (eds), pp. 53–69. Lippincott: Philadelphia

Celis E, Van Tsai A, Crimi C, DeMars R, Wentworth PA, Chesnut RW, Grey HM, Sette A and Serra HM (1994) Induction of anti-tumour cytotoxic T lymphocytes in normal humans using primary cultures and synthetic peptide epitopes. *Proc Natl Acad Sci USA* **91**: 2105–2109

Colombo MP and Forni G (1994) Cytokine gene transfer in tumour inhibition and tumour therapy: where are we now? *Immunol Today* **15**: 48–51

Coulie P, Somville M, Lehmann F, Hainaut P, Brasseur F, Devos R and Boon T (1992) Precursor frequency analysis of human cytolytic T lymphocytes directed against autologous melanoma cells. *Int J Cancer* **50**: 289–297

Culver KW (1996) Measuring success in clinical gene therapy research. *Mol Med Today* **2**: 234–236

Decker T and Lohmann-Matthes ML (1988) A quick and simple method for quantitation of lactate dehydrogenase release in measurements of cellular cytotoxicity and tumour necrosis factor (TNF) activity. *J Immunol Methods* **15**: 61–69

Fearon ER, Pardoll DM, Itaya T, Golumbek P, Livitsky HI, Simons JW, Karasuyama H, Vogelstein B and Frost P (1990) Interleukin-2 production by tumour cells bypasses T helper function in the generation of an antitumor response. *Cell* **60**: 397–403

Ferrone S (1994) Melanoma, immune surveillance, and immunotherapy. *J Clin Invest* **93**: 1351–1352

Filion MC, Bradley AJ, Devine DV, Decary F and Chartrand P (1995) Autoreactive T cells in healthy individuals show tolerance in vitro with characteristics similar to but distinct from clonal anergy. *Eur J Immunol* **25**: 3123–3127

© Cancer Research Campaign 1998

Finke S, Grimm B, Möller P, Schadendorf D, Neubauer A, Huhn D and Schmidt-Wolf I (1997) Increase of cytotoxic sensitivity of primary melanoma cells transfected with the interleukin-7 gene to autologous and allogeneic immunologic effector cells. *Cancer Gene Ther* (in press)

Gansbacher B, Houghton AN, Livingston P, Minasian L, Rosenthal F, Gilboa E, Golde D, Oettgen H, Steffen T, Yang SY and Wong G (1992) A pilot study of immunisation with HLA-A2 matched allogeneic melanoma cells that secrete interleukin-2 in patients with metastatic melanoma. *Hum Gene Ther* **3**: 677–690

Herr W, Wölfel T, Heike M, Meyer zum Büschenfelde KH and Knuth A (1994) Frequency analysis of tumour-reactive cytotoxic T lymphocytes in peripheral blood of a melanoma patient vaccinated with autologous tumour cells. *Cancer Immunol Immunother* **39**: 93–99

Hickman CJ, Crim JA, Mostowski HS and Siegel JP (1990) Regulation of human cytotoxic T lymphocyte development by IL-7. *J Immunol* **145**: 2415–2420

Ho VC and Sober AJ (1990) Therapy of cutaneous melanoma: an update. *J Am Acad Dermatol* **22**: 159–176

Hock H, Dorsch M, Kunzendorf U, Zhihai Q, Diamantstein T and Blankenstein T (1993) Mechanisms of rejection induced by tumour cell-targeted gene transfer of interleukin 2, interleukin 4, interleukin 7, tumour necrosis factor, or interferon gamma. *Proc Natl Acad Sci USA* **90**: 2774–2778

Houghton AN (1994) Cancer antigens: immune recognition of self and altered self. *J Exp Med* **180**: 1–7

Kawakami Y, Eliyahu S, Delgado CH, Robbins PF, Sakaguchi K, Appella E, Yannelli JR, Adema GJ, Miki T and Rosenberg SA (1994) Identification of a human melanoma antigen recognised by tumour-infiltrating lymphocytes associated with in vivo tumour rejection. *Proc Natl Acad Sci USA* **91**: 6458–9462

Kos FJ and Müllbacher A (1992) Induction of primary anti-viral cytotoxic T cells by in vitro stimulation with short synthetic peptide and interleukin-7. *Eur J Immunol* **22**: 3183–3185

Mackensen A, Carcelain G, Viel S, Raynal M-C, Michalaki H, Triebel F, Bosq J and Hercend T (1994) Direct evidence to support the immunosurveillance concept in a human regressive melanoma. *J Clin Invest* **93**: 1391–1402

Miller AR, McBride WH, Dubinett SM, Dougherty GJ, Thacker JD, Shau H, Kohn DB, Moen RC, Walker MJ, Chui R, Schuck BL, Rosenblatt JA, Huang M, Dhanani S, Rhoades K and Economou J (1993) Transduction of human melanoma cell lines with the human interleukin-7 gene using retroviral-mediated gene transfer: comparison of immunological properties with interleukin-2. *Blood* **82**: 3686–3694

Namen AE, Schmierer AE, March CJ, Overell RW, Park LS, Urdal DL and Mochizuki DY (1988) B cell precursor growth-promoting activity. *J Exp Med* **167**: 988–1002

Naume B and Espevik T (1991) Effects of IL-7 and IL-2 on highly enriched CD56+ natural killer cells. *J Immunol* **147**: 2208–2214

Oettgen HF and Old LJ (1991) The history of cancer immunotherapy. In *Biologic Therapy of Cancer, Principles and Practice*, deVita VT, Helman S and Rosenberg SA. (eds), p. 87. Lippincott: Philadelphia

Old LJ (1981) Cancer immunology: the search for specificity – GHA Clowes Memorial Lecture. *Cancer Res* **41**: 365–370

Pardoll DM (1995) Paracrine cytokine adjuvants in cancer immunotherapy. *Annu Rev Immunol* **13**: 399–415

Robbins PF, El-Gamil M, Kawakami Y and Rosenberg SA (1994) Recognition of tyrosinase by tumour-infiltrating lymphocytes from a patient responding to immunotherapy. *Cancer Res* **54**: 3124–3126

Schadendorf D, Worm M, Algermissen B, Kohlmus CM and Czarnetzki BM (1994) Chemosensitivity testing of human malignant melanoma – a retrospective analysis of clinical response and in vitro drug sensitivity. *Cancer* **73**: 103–108

Schadendorf D, Makki A, Stahr C, Van Dyck A, Wanner R, Scheffer GL, Flens MJ, Scheper R and Henz BM (1995) Membrane transport proteins associated with drug resistance expressed in human melanoma. *Am J Pathol* **147**: 1545–1552

Schadendorf D, Czarnetzki BM and Wittig B (1995) Interleukin-7, interleukin-12, and GM-CSF gene transfer in patients with metastatic melanoma. *J Mol Med* **73**: 473–477

Schadendorf D, Fichtner I, Makki A, Alijagic S, Küpper M, Mrowietz U and Henz BM (1996) Metastatic potential of human melanoma cells in nude mice – characterisation of phenotype, cytokine secretion and tumour-associated antigens. *Br J Cancer* **73**: 194–199

Schmidt-Wolf GH and Schmidt-Wolf IGH (1995) Cytokines and cytokine gene therapy. *Eur J Immunol* **25**: 1137–1140

Schroff M, Schinagl M, Ertz S, Takeya M, Brendel S, Dorbic T, Möller P, Schadendorf D and Wittig B (1997) Ballisto-magnetic transfer of expression vectors into human cell nuclei, ex vivo, and its application in cancer gene therapy. (submitted)

Taswell C (1981) Limiting dilution assay for the determination of immunocompetent cell frequencies. I. Data analysis. *J Immunol* **126**: 1614–1619

Tepper RI, Pattengale PK and Leder P (1989) Murine interleukin 4 displays potent anti-tumour activity in vivo. *Cell* **57**: 503–512

Vieweg J and Gilboa E (1995) Considerations for the use of cytokine-secreting tumour cell preparations for cancer treatment. *Cancer Treat Rev* **13**: 193–201

*© Cancer Research Campaign 1998*